# An integrative investigation on significant mutations and their down-stream pathways in lung squamous cell carcinoma reveals CUL3/KEAP1/NRF2 relevant subtypes

**DOI:** 10.1186/s10020-020-00166-2

**Published:** 2020-05-20

**Authors:** Zongang Liu, Meiyan Deng, Lin Wu, Suning Zhang

**Affiliations:** Department of Thoracic Surgery, Shengjing Hospital of China Medical University, No.36 Sanhao Street, Heping District Shenyang, Liaoning 110004 People’s Republic of China

**Keywords:** Lung squamous cell carcinoma, Down-stream pathways, *NFE2L2*, *KEAP1*, Prognosis, Subtypes

## Abstract

**Background:**

Molecular mechanism of lung squamous cell carcinoma (LUSC) remains poorly understood, hampering effective targeted therapies or precision diagnosis about LUSC. We devised an integrative framework to investigate on the molecular patterns of LUSC by systematically mining the genomic, transcriptional and clinical information.

**Methods:**

We utilized the genomics and transcriptomics data for the LUSC cohorts in The Cancer Genome Atlas.. Both kinds of omics data for 33 types of cancers were downloaded from The NCI’s Genomic Data Commons (GDC) (https://gdc.cancer.gov/about-data/publications/pancanatlas). The genomics data were processed in mutation annotation format (maf), and the transcriptomics data were determined by RNA-seq method. Mutation significance was estimated by MutSigCV. Prognosis analysis was based on the cox proportional hazards regression (Coxph) model.

**Results:**

Significant somatic mutated genes (SMGs) like *NFE2L2*, *RASA1* and *COL11A1* and their potential down-stream pathways were recognized. Furthermore, two LUSC-specific and prognosis-meaningful subtypes were identified. Interestingly, the good prognosis subtype was enriched with mutations in CUL3/KEAP1/NRF2 pathway and with markedly suppressed expressions of multiple down-stream pathways like epithelial mesenchymal transition. The subtypes were verified by the other two cohorts. Additionally, primarily regulated down-stream elements of different SMGs were also estimated. *NFE2L2*, *KEAP1* and *RASA1* mutations showed remarkable effects on the subtype-determinant gene expressions, especially for the inflammatory relevant genes.

**Conclusions:**

This study supplies valuable references on potential down-stream processes of SMGs and an alternative way to classify LUSC.

## Background

Lung cancer is one of the most frequent malignant neoplasms and one major cause of cancer death around the world (Torre et al. 2016; Malhotra et al. 2016). It is a highly heterogeneous and complex disease and there are many subtypes. Non-small cell lung cancer (NSCLC) is the most common lung cancer type, which can be mainly divided into lung adenocarcinoma (LUAD) and lung squamous cell carcinoma (LUSC) (Derman et al. 2015). Several targeted drugs have been developed to treat LUAD patients which have mutations on specific genes like *EGFR* (Paez et al. 2004) and *ALK* (Felip et al. 2011), and have displayed remarkable therapeutic effects. However, these drugs were not applicable to the LUSC patients since the specific mutations were rarely observed in LUSC. LUSC differs from LUAD in both pathological and molecular levels. Some genomic studies have revealed significant mutations in a collection of genes, such as *TP53*, *PIK3CA*, *NFE2L2*, *KEAP1*, *FBXW7*, etc., and some of the mutations showed significant associations with LUSC prognostic outcomes (Choi et al. 2017; Cancer Genome Atlas Research N 2012).

Simply identification of the significant mutations is not sufficient to describe the complicated molecular mechanism of LUSC. Each mutation can lead to direct or in-direct effects on cascades of down-stream processes. For instance, NRF2 (protein encoded by *NFE2L2*) mainly activates cellular antioxidant responses by transcriptional regulation of numerous cytoprotective genes which can combat harmful effects such as xenobiotics and oxidative stress (Wu et al. 2019). Besides, NRF2 has also been demonstrated to regulate mTOR signaling pathway (Bendavit et al. 2016) and inflammatory response (Kobayashi et al. 2016). Some studies have discovered the dual roles of NRF2 in cancer (Wu et al. 2019; Lau et al. 2008; Gonzalez-Donquiles et al. 2017). It is unquestionable there are a great deal of un-revealed down-stream pathways underlying the driven mutations. Consequently, to better understand the molecular mechanism and to design effective personalized treatments for LUSC, a comprehensive understanding about both the SMGs as well as their potential down-stream effects is essential.

Here, we put forward a systematical study to investigate on both the significant mutations and their down-stream pathways by integratively mining the genomic, transcriptional and clinical data of LUSC cohorts. Meanwhile, we also attempt to examine whether the differential expression profiles of down-stream pathways can help identify clinical meaningful LUSC subtypes. As results, we identified two LUSC-specific subtypes which showed significant differences in mutational, expressional as well as clinical patterns, and the better prognosis subtype was enriched by mutations in CUL3/KEAP1/NRF2 pathway and displayed suppressed expressions of genes involved in epithelial mesenchymal transition (EMT), inflammatory responses and other potential down-stream pathways.

## Materials and methods

### TCGA data preparing

We mainly utilized the genomics and transcriptomics data for the LUSC cohorts in the Cancer Genome Atlas (TCGA) (Cancer Genome Atlas Research N 2012). Both kinds of omics data were downloaded from The NCI’s Genomic Data Commons (GDC) (https://gdc.cancer.gov/about-data/publications/pancanatlas) which contained multi-omics data resources for 33 types of cancers. The genomics data were processed in mutation annotation format (maf), and the transcriptomics data were determined by RNA-seq method. From this pan-cancer atlas, we mainly extracted the data corresponding to LUSC patients for this study. Besides, we also extracted the transcriptomics and clinical data for LUAD patients for independent comparison.

### Pathway information

Pathway information, i.e., pathway names as well as genes belonging to each pathway, were obtained from Molecular Signatures Database (MsigDB, http://software.broadinstitute.org/gsea/msigdb) (Liberzon et al. 2015), where the hallmark gene sets were utilized for the pathway-based analyses.

### Identification of significant somatic mutated genes (SMGs)

For the maf mutation file, we utilized MutSigCV (version 1.3.4) (Lawrence et al. 2013) to recognize significant SMGs and the significance threshold was set as q-value < 0.1. Then, we utilized the R package maftools to visualize the mutation information of these significant SMGs for all TCGA LUSC patients. Besides, we applied pair-wise Fisher’s Exact test to detect mutually exclusive or co-occurring SMGs.

### Identification of potential downstream genes of SMGs

The RNA-seq based transcriptomics data were preprocessed based on the voom algorithm (Law et al. 2014) in the R package limma (Ritchie et al. 2015). Next, for each SMG, we separated the samples into mutated and wild type sets, and utilized T-test (unpaired, two-sided) to identify which genes were differentially expressed between mutated and wild type set in the transcriptomics data, then genes with FDR less than 0.1 were taken as the potential downstream genes of the SMG.

### Survival analysis based on gene expression levels

The clinical information of all TCGA-LUSC patients was also obtained from the GDC. For the SMG relevant potential downstream genes, we also analyzed their prognosis impacts. For each such gene, we utilized the Cox proportional hazards (coxph) regression model in the R package “survival” (Therneau and Grambsch 2000) to examine whether the expression level of this gene has a significant influence on the survival rate. According to the coxph results, genes with *p*-values less than 0.05 were regarded as prognosis-relevant, and if the regression coefficients are larger than 0, then higher expression levels will correspond to worse survival rates, otherwise, higher expression levels will correspond to better survival rates.

### Identification of potential down-stream pathways of SMGs

For each individual SMG, we separated the samples into mutated and wild type sets and calculated the difference value of the mean mRNA expression levels of each gene between the two groups. Then, we ranked the genes according to the difference values, utilized the ranked gene list as input for Gene Set Enrichment Analysis (GSEA) (Subramanian et al. 2005), and obtained the *p*-values. At last, pathways with p-values less than 0.01 were regarded as the potential down-stream pathways of the SMG, and the -log10(p) was calculated as the SMG-pathway relevant score.

### Unsupervised clustering of patients based on SMG relevant genes and pathways

The mRNA-level expression matrix of solid tumor tissues in terms of all SMGs and all gene members in their down-stream pathways were utilized as the input for clustering analysis. This expression matrix was scaled by subtracting the mean level and being divided by the standard derivation with respect to each individual gene. Based on the scaled expression matrix, we applied a consensus clustering method implemented in the R package “ConsensusClusterPlus” (Wilkerson and Hayes 2010) where the basic clustering method was set as “partitioning around medoids” to cluster the patients into 2 clusters.

### Evaluate the importance of genes for the clustering results

After clustering the patients into 2 clusters, we used the random forest (Liaw and Wiener 2002) algorithm to evaluate the importance of all genes in the input expression matrix for predicting the accurate cluster labels. These genes were ranked by the importance score. Besides, we also examined enrichment significance of the top-50 important genes in each pathway based on the hypergeometric distribution.

### Validating the prognosis differences based on the other independent lung cancer cohorts

The mRNA expression matrix and the corresponding clinical information for two lung cancer cohorts (GSE30219 and GSE37745) were downloaded from Gene Expression Omnibus (GEO) database by the R package ‘GEOquery’ (Sean and Meltzer 2007). We constructed a cluster-label predictor based on the TCGA-LUSC expression matrix of the shared genes between the top-50 important and genes measured in the GEO dataset. This predictor was trained by a generalized linear model (implemented by the R package “glmnet” (Simon et al. 2011)). We used this predictor on the GEO datasets or TCGA-LUAD cohort to predict the corresponding cluster-labels for each patient, and survival differences of the two predicted clusters were tested by log-rank and the corresponding survival curves were estimated by the Kaplan-Meier method.

### Statistical analysis

All statistical analysis and computations were performed in R. The detailed statistical methods were included in the corresponding sections.

## Results

### Significant SMGs in LUSC rarely generate significant prognostic impacts themselves

**Fig. 1:**
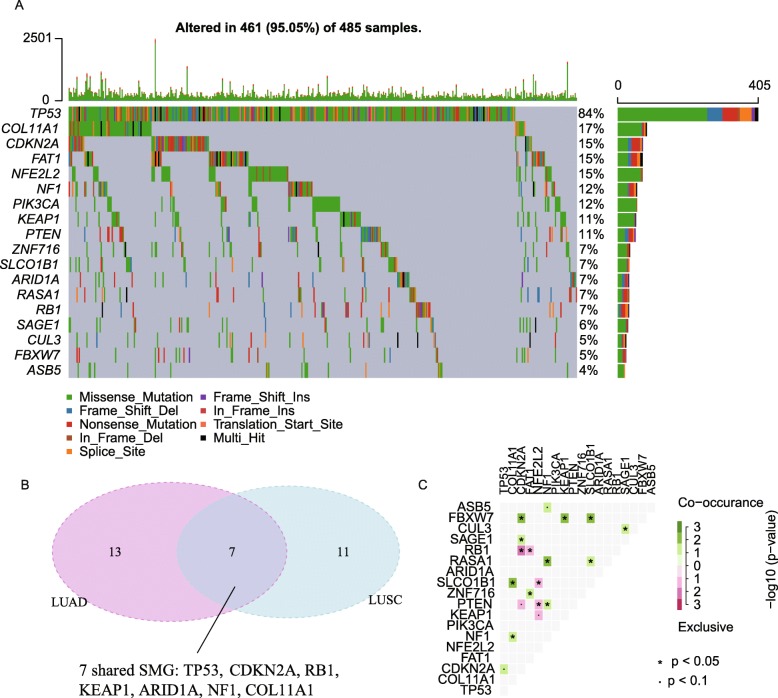
Significant somatic gene mutations in TCGA LUSC. **a**. Oncoplot of SMGs in LUSC. **b**. Overlapped SMGs between LUSC and LUAD. **c**. Interactions within the SMGs

**Fig. 2:**
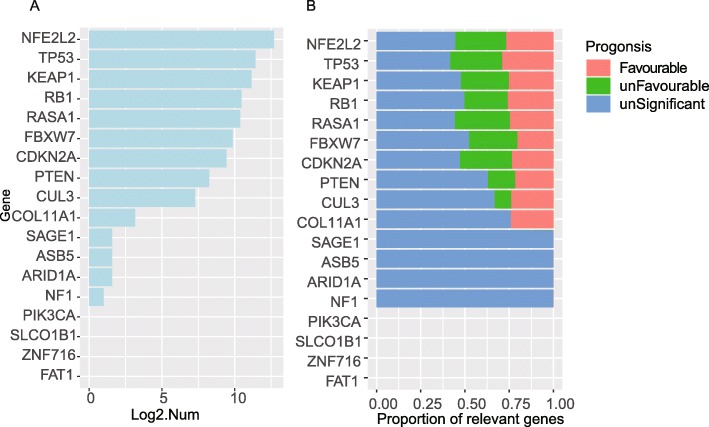
Somatic mutations generate remarkable down-stream alterations. **a**. The number of SMG relevant genes which were differentially expressed between samples with and without certain mutation. **b**. The proportion of SMG relevant genes with different prognostic effects

According to the gene mutation data of 502 LUSC patients in TCGA, we identified 18 potential SMGs by MutSigCV (q-value < 0.1, Fig. 1a). The mutations for most of these SMGs like *TP53*, *CDKN2A*, *KEAP1*, *PTEN*, *PIK3CA*, *NFE2L2*, *RB1*, etc. have already been identified in previous studies (Cancer Genome Atlas Research N 2012). Compared to the SMG identified for LUAD from TCGA, there are only 7 common SMGs including *TP53*, *CDKN2A*, *RB1*, *KEAP1*, *ARID1A*, *NF1*, *COL11A1* between LUSC and LUAD (Fig. 2b). Some SMGS were specific to one type of cancer. The significant SMGs like *NFE2L2* and *RASA1* are rarely mutated in LUAD patients. These results confirmed the differential molecular mechanism between LUAD and LUSC.

To evaluate the most direct mutational effects, we compared the expressions of these SMGs in the mutated and wild type samples. We found that some of the SMGs can lead to significant expressional alterations. For instance, the mutations of RB1 were related with significantly reduced expressions of RB1 while mutations of *CDKN2A*, *NFE2L2* might improve the expression levels (Figure S1A). CDKN2A is a well-known tumor suppressor gene in lung cancer, and it is frequently inactivated in LUSC (Wikman and Kettunen 2006). Here, we found its mutation may be related with a higher level of expression in this gene, then the inactivation of CDKN2A may be caused by some other effects like silencing methylation or homozygous deletion (Cancer Genome Atlas Research N 2012; Wikman and Kettunen 2006). Besides, only two of the expressional alterations (*KEAP1* and *NFE2L2*) can lead to significant prognosis influence (Figure S1B).

Among the significant SMGs for LUSC, the mutations of certain pairs of SMGs, like *NFE2L2* and *KEAP1*, *RB1* and *CDKN2A*, *PTEN* and *CDKN2A* were mutual exclusive (Fig. 1c), suggesting the potential convergent effects on the same downstream elements between different SMGs. For instance, *KEAP1* encodes the adapter protein of an E3 ligase complex which can ubiquitinate NRF2, and previous studies have proven that mutations in *KEAP1* and *NFE2L2* may lead to NRF2 activation which may further contribute to spontaneous cancer development (Leinonen et al. 2014; Taguchi et al. 2010).

### Somatic mutations generate remarkable down-stream alterations

To reveal the down-stream influences generated by the SMGs in a more comprehensive perspective, we evaluated the mutation effects of all SMGs in a genome-wide manner. For each individual SMG, we identified the genes with significant expressional alterations between samples with and without mutations in terms of this SMG. According to this results, *NFE2L2* was with the largest number of significantly altered down-stream genes (false discover rate [FDR] adjusted *p* value < 0.1, T-test, unpaired, two-sided), *TP53*, *RB1*, *KEAP1* and *RASA1* were among the top-5 (Fig. 2a). The mutations of these SMGs might lead to significant expressional alterations of the other genes. Based on the top-ranked (the ranking is based on the fold change of mean expression levels between mutated and wild type samples) expressional altered genes, a network was constructed (Figure S2). It described the potential down-stream regulated genes for the SMGs. Interestingly, we found that *NFE2L2* and *KEAP1*, *RB1* and *CDKN2A*, two pairs of SMGs which showed co-exclusive mutation patterns in Fig. 1c, were tightly connected by the shared potential down-stream genes also (Figure S2). This is just coherent with the above notion that co-exclusive SMGs may produce convergent down-steam effects. Meanwhile, some SMGs may lead to remarkable expressional alterations of well-known oncogenes or tumor suppressor genes, e.g., the mutations in RB1 may regulate the up-regulation of the oncogene DEK, and the mutations in TP53 may be related with up-regulation of both oncogene SOX2 and tumor suppressor gene WNK2 (Figure S2, role of the downstream genes were annotated based on the COSMIC database (Forbes et al. 2017)), suggesting the multi-aspect downstream impacts of the SMGs. Besides, some of the down-stream genes can be directly regulated by the mutated genes. For instance, NFE2L2 is a transcriptional factor that binds with the antioxidant response element (ARE), and among the top-ranked down-stream genes (Figure S2), NQO1 contains the ARE motif in the gene promoter, and has been reported as a direct target of NFE2L2 (Dhakshinamoorthy and Jaiswal 2000).

Meanwhile, we also evaluated the prognostic effects of all the potential down-steam genes. For top-8 ranked SMGs in terms of the number of potential down-stream genes (Fig. 2b), nearly half of these potential down-steam genes were with significant prognosis (log-rank *p* < 0.05) impacts, and almost half favourable (higher expressions will correspond to a better survival rate) and half un-favourable (higher expressions will correspond to a worse survival rate). For *PTEN*, *CUL3*, and *COL11A1*, the portions of prognosis relevant potential down-stream genes were much lower, and the favourable ones took the majority. The distinct prognosis impacts for these potential down-steam genes of the same SMGs or across different SMGs both suggested that the complexity of the down-stream impacts generated by the SMGs in LUSC.

### Potential down-stream pathways influenced by the SMGs

**Fig. 3:**
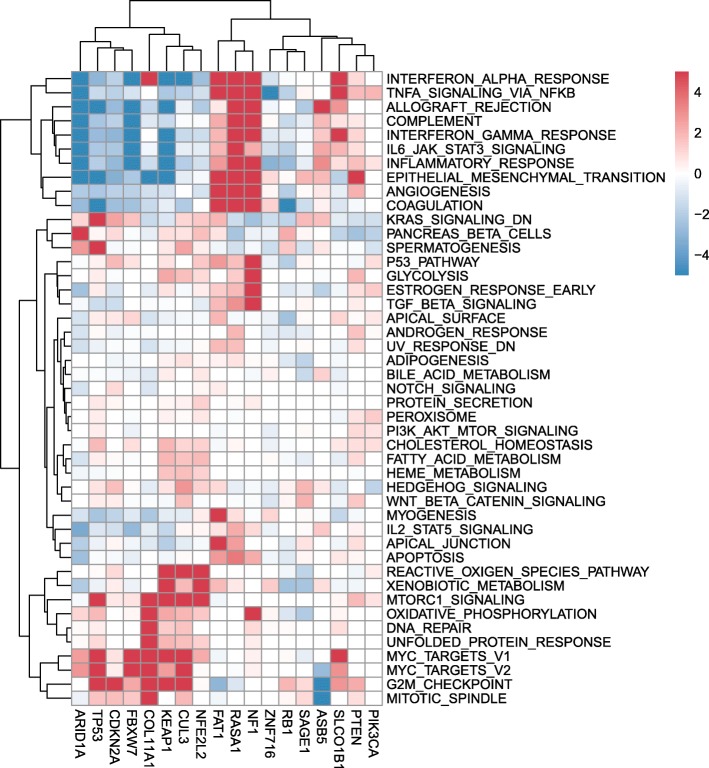
Heatmap about the potential down-stream pathways of the SMGs. GSEA is performed to evaluation the significance of each pathway. Each cell is colored according to the transformed GSEA-based FDR value where the absolute value is equal to the –log10(FDR) and the sign represents the regulation direction (+/−, up/down regulation)

Furthermore, we also recognized the potential down-steam pathways of each SMG based on the genome-wide expressional alterations. According to the clustering results of the SMG-pathway relevance scores, two major SMG clusters or groups emerged (Fig. 3). The first group included *ARID1A*, *TP53*, *CDKN2A*, *FBXW7*, *NFE2L2*, *CUL3*, *KEAP1* and *COL11A1*, their mutation may lead to the up-regulations of mTOR signaling pathway, MYC targets and the down-regulations of inflammatory response. The second one included *FAT1*, *RASA1*, *NF1*, *ZNF716*, *RB1*, et al., and their mutations may generate somewhat reversed pathway impacts compared to the first group, for instance, their mutations may largely lead to up-regulations in inflammatory response and rarely lead to significant alterations in the mTOR signaling pathway. The SMG-pathway relevance imply the convergent effects among certain SMGs, like *NFE2L2*, *KEAP1* and *CUL3* where both KEAP1 and CUL3 are components of E3 ligase complex and NRF2 is the targeted ubiquitination substrate (Leinonen et al. 2014). Meanwhile, contrary regulation patterns between some SMGs, like KEAP1 and RASA1 were also observed. Without a global perspective of the down-stream influences of SMGs, it is impossible to understand the complex molecular mechanism of LUSC development and progress.

### Expressional profiles of the potential downstream pathways help reveal two subtypes with significant differences in SMGs and prognosis outcomes

**Fig. 4:**
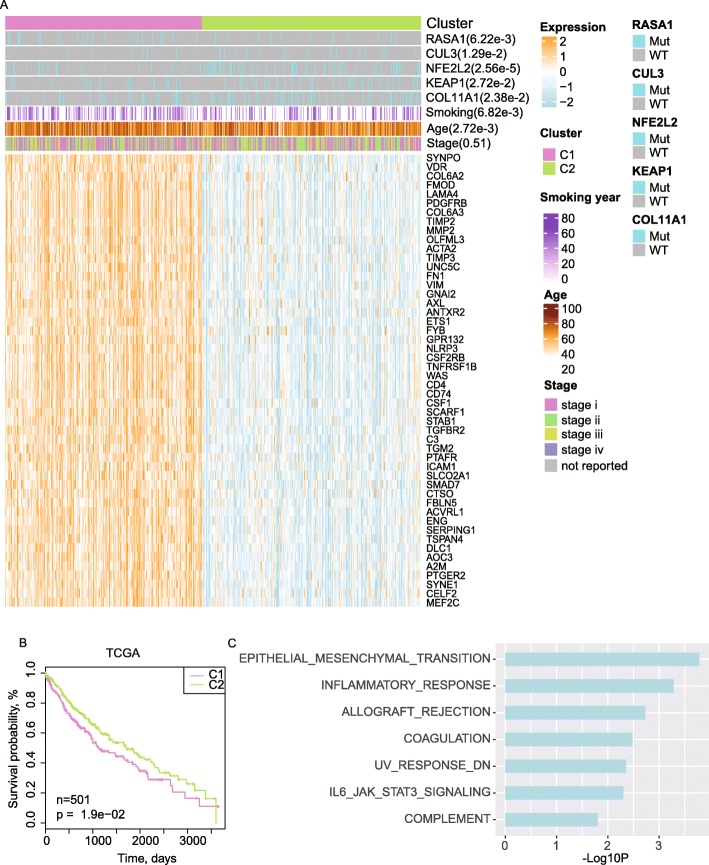
Clinical influences of the somatic mutations together with relevant pathways. **a** Clustering the LUSC patients by their expression profiles in both the significant mutated genes and other genes in the relevant pathways. **b** Kaplan-Meier (KM)-plot of the 10 year overall survival curves for two clusters of patients. **c** Enriched pathways of the top-50 important genes for stratifying the cohort into the two clusters. The gene importance was scored by random forest method

To obtain a more holistic view of the molecular and clinical heterogeneity, we separated the TCGA LUSC patients into two clusters based on the expression matrix with respect to all the SMGs and their down-stream pathways (Fig. 4a, see materials and methods). Comparing these two clusters, we observed that a collection of EMT relevant genes including *TGM2* (Ma et al. 2018), *FBLN5* (Lee et al. 2008), *PDGFRB* (Chang et al. 2018), etc. and a series of genes involved in the inflammatory response pathway including *PTAFR* (Nakamura et al. 1991), *ICAM1* (van Buul et al. 2007), *GPR132* (Lin and Ye 2003), etc. were up-regulated in Cluster 1 (C1) comparing to Cluster 2 (C2). The significantly differential expression patterns were probably associated with the mutations of *NFE2L2*, *CUL3*, *KEAP1*, *COL11A1* and *RASA1*, where mutations of *NFE2L2*, *CUL3*, *KEAP1* and *COL11A1* were significantly enriched in C2 (*p* < 0.05, hypergeometric distribution), while *RASA1* mutations were enriched in C1 (*p* = 6.22e-3, hypergeometric distribution). The mutations of these five SMGs together with their significant influences on the down-stream pathways may contribute to the LUSC molecular heterogeneity.

Notably, the two differential groups displayed a significant difference in prognosis, where C1 showed significantly lower survival rate than C2 (Fig. 4b, *p* < 0.05). Besides, the poor prognosis of C1 may also be related with a significantly longer time of smoking history and older ages among the patients (Wilcox-test, two-sided, unpaired, *P*-value = 6.82e-3 and 2.72e-3 for smoking year and age respectively), however, the identified two clusters were totally different from the original tumor stage definition (Chi-squared test, *P* = 0.51). Interestingly, although previous studies have revealed that *KEAP1*/*NEF2L2* mutations may contribute to LUSC or poor prognosis (Tian et al. 2016; Cloer et al. 2019; Solis et al. 2010), here, we found that both *KEAP1* and *NFE2L2* mutations were enriched in the better prognosis subtype (C2, Fig. 4a and b). This is probably in part because the dual-role of NRF2 in cancer (Wu et al. 2019) and it also indicates that although certain mutations may be driven factors for LUSC, their prognostic impacts can be conditional rather than absolutely un-favourable. Meanwhile, examinations on the prognostic impacts of the subtype relevant SMGs showed each individual SMG cannot lead to significant survival differences (Figure S3). The cancer subtypes and their underlying prognosis difference were not determined by certain SMGs, but a combination of multiple factors like smoking history as well as the combined down-stream effects.

The molecular level heterogeneity of LUSC is reflected in numerous SMGs and pathways. In addition to the EMT and inflammatory response pathways, the highly-important genes for separating the LUSC patients into the two clusters were also significantly enriched in the UV-response, IL6-JAK-STAT3 signaling and TGF-Beta signaling pathways (Fig. 4c).

### Robustness of the two subtypes was verified by the other independent LUSC cohorts

**Fig. 5:**
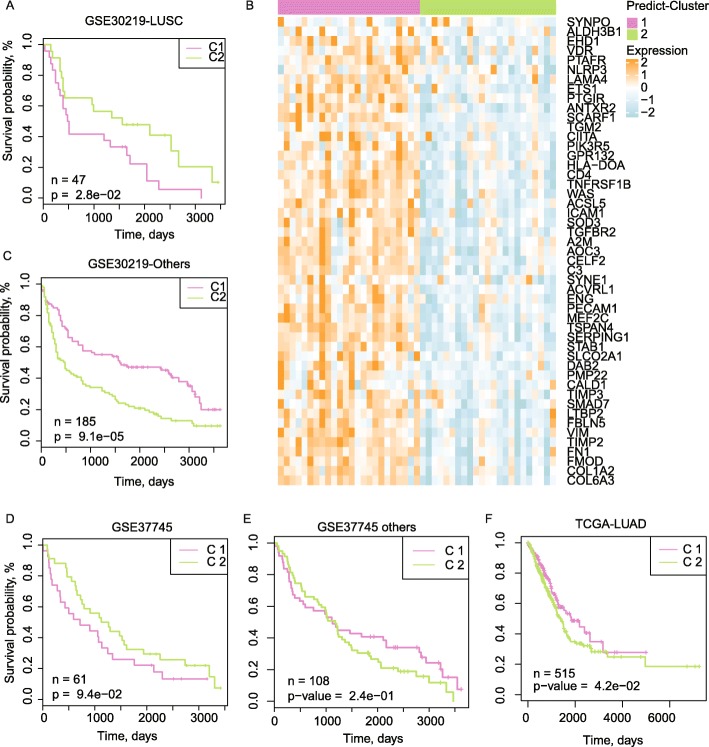
Validation of the two clusters by an independent lung cancer dataset. **a**-**b**. KM plots of the survival curves for two predicted patient clusters in GSE30219 where only LUSC patients (**a**) or the other types of lung cancer patients (**b**) were taken into consideration. **c**. Expression profiles of the important genes. **d**-**e**. KM-plots of the survival curves for two predicted subtypes in GSE37745 where only LUSC patients (**a**) or the other types of lung cancer patients were considered. (**f**) KM-plots of the survival curves for two predicted subtypes in the TCGA-LUAD cohort

To validate whether the identified expressional and prognostic differences in the two subtypes also exist in the other LUSC cohorts, we constructed a subtype predictor based on the expressional profiles and clustering results of the TCGA-LUSC cohorts and applied it to predict the corresponding cluster labels for the LUSC patients in GEO datasets. For GSE30219, two cluster patients annotated by this framework were also with significant prognostic differences (Fig. 5a), just as observed in TCGA-LUSC, and similar expressional patterns emerged (Fig. 5b). To be noted, when the other types of lung cancer were taken into consideration, the corresponding clusters were with totally reversed prognosis outcomes (C1 were with better survival rates, Fig. 5c), suggesting the specificity of the identified LUSC subtypes. To examine the robust of the LUSC-based heterogeneity pattern further, we also applied the cluster predictor on the other lung cohorts, the identified two LUSC clusters also displayed similar prognostic differences as observed in TCGA-LUSC (Fig. 5d), but the prognosis differences disappeared (Fig. 5e) or showed opposite outcomes (Fig. 5f) for the other non-LUSC lung cancers. This result suggested the *NFE2L2*, *KEAP1* and *RASA1* relevant expressional heterogeneity and the corresponding prognostic influences mainly hold true in LUSC patients, but not for the other types of lung cancers.

### Identification of the primarily regulated genes among the potential down-stream pathways of different SMGs

**Fig. 6:**
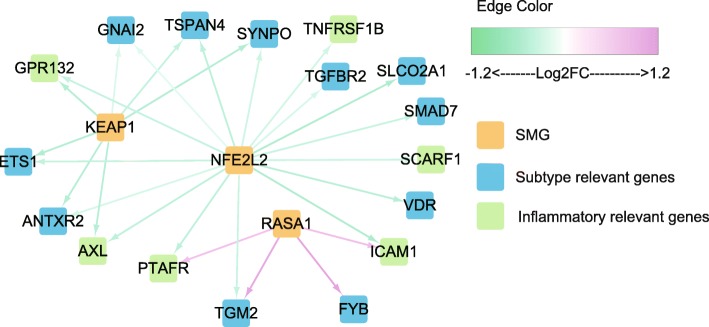
A core association network between SMG and down-stream elements. Yellow nodes are SMGs, the other nodes are genes showing the top-50 important scores in separating the two subtypes for LUSC, and the inflammatory relevant ones are marked by green. Edges stand for the significant associations (T-test, un-paired, two-sided, FDR adjusted *P*-value < 0.05), and edge colors represent the log2 transformed fold change (log2FC) values of down-stream genes in mRNA level between samples with and without mutations in the SMG

To further understand the relationships between mutational patterns and down-stream expressional profiles which all contributed to the two identified subtypes, we further examined whether the mutations of the five SMGs were associated with significant expressional alterations of the top-50 important genes in separating two subtypes. Therefore, we constructed a core SMG-gene association network (Fig. 6) describing which genes involved in the down-stream pathways were more likely to be directly regulated by the SMGs. As results, *NFE2L2* mutations showed significant influences on the largest number of subtype-determinant down-stream genes, especially the genes in inflammatory response pathway, e.g., *ICAM1* (van Buul et al. 2007), *GPR132* (Lin and Ye 2003), *AXL* (Bottai et al. 2016), *SCARF1* (Son et al. 2015), etc. Additionally, *KEAP1* and *RASA1* also showed significant effects on certain *NFE2L2*-relevant genes (e.g., *ANTXR2*, *GNAI2*, *PTAFR* and *TGM2*), suggesting the cooperative regulating modes among the SMGs. This core SMG-gene network only covers a part of the top-50 important genes (17 out of 50), implying the differentially expressional patterns between two subtypes were not simply caused by individual mutations, but the results of complicated and multi-factorial perturbations.

## Discussion

The accumulation of various types of omics data has promoted the understanding about molecular mechanism of cancers. The initial analysis on the TCGA-LUSC cohorts covered 178 samples (Cancer Genome Atlas Research N 2012). With the completion of the TCGA project (Cancer Genome Atlas Research et al. 2013), more LUSC samples were accumulated and measured and more comprehensive omics data were provided. Based on the larger scale of LUSC data resource, we re-analyzed the mutation and RNA-seq data, identified the SMGs and their potential down-stream genes and pathways, and revealed an alternative way to classify LUSC subtypes which showed significant prognostic and molecular differences across multiple cohorts consistently.

Cancer is a complex disease. Frequent SMGs were taken as potential therapeutic targets, however, only specific patients are suitable for certain forms of therapeutic strategies. Meanwhile, many SMGs were un-druggable. A comprehensive understanding about the down-stream processes of the SMGs can help improve the efficiency of precision medicine and provide alternative therapeutic targets. According to our analysis, we identified 18 SMGs with significant mutations in LUSC. These SMGs, especially *NFE2L2*, *TP53* and *RASA1* were related with the expressional alterations of large number of genes. The activity of NFE2L2 is known to be mainly regulated via its interaction with KEAP1 (Bryan et al. 2013). Here, we observed that mutations of *NFE2L2* and *KEAP1* were highly co-exclusive, suggesting the convergent down-stream effects. A SMG down-stream gene analysis showed that the down-stream functions of all SMGs where both *NFE2L2* and *KEAP1* exhibited significant influences on the pathways of mTOR signaling and inflammation responses (Fig. 3) which have been reported (Bendavit et al. 2016; Kobayashi et al. 2016).

Nearly half of the potential down-stream regulated genes can generate significant prognostic impacts. However, the expressional alterations underlying the same SMG can also lead to opposite prognostic effects. The complex molecular features of LUSC make it impossible to predict the prognosis with respect to single SMG or its relevant down-stream pathways. Here, to obtain a more comprehensive perspective, we recognized two LUSC subtypes (termed C1 and C2) by clustering the TCGA-LUSC patients according to the expression profiles of all SMGs and their relevant pathways. The two subtypes showed distinctively mutational patterns in *NFE2L2*, *KEAP1*, *RASA1*, *CUL3* and *COL11A1*, and remarkably differential expressions of genes involved in multiple pathways like EMT, inflammatory response, and IL6-JAK-STAT3 signaling pathways. These molecularly differential subtypes also showed significantly prognosis differences. Interestingly, the better survival subtype (C2) was observed to be with higher mutation frequencies considering *NFE2L2*, *KEAP1* and *CUL3* which may be onco-driver of LUSC (Pölönen and Levonen 2016; Kandoth et al. 2013). This indicates although mutations of certain genes may contribute to the development of cancer, their mutations are not indicators of poor prognosis. This may also be related with the dual roles of NRF2 (Lau et al. 2008; Gonzalez-Donquiles et al. 2017). In any case, the prognostic differences between subtypes are determined by a series of factors but not only one simple condition like a SMG or over-expressed gene. Accordingly, a subtype predictor was trained and tested based on the expressional profiles of LUSC patients. Similar subtypes were also recognized in the other independent LUSC cohorts where patients with C1-like expressional profiles were also with worse survival rates. However, when applied on LUAD, C1-like and C2-like patients were with opposite prognosis outcomes than that observed in LUSC.

Furthermore, primely regulated pathway members of the subtype-relevant SMGs were also recognized in this study. *NFE2L2*, *KEAP1* and *RASA1* were more likely to have direct influences on some key down-stream elements in a cooperative way, especially for those belonging to inflammatory response pathway. These potential down-stream pathways for *KEAP1*/*NFE2L2*/*CUL3* mutations can also provide alternative way for LUSC treatment where therapeutic interventions for governing NRF2 activity have proven largely intractable.

However, there are some limitations of this study. Here, for the gene mutation analysis, we only focused on gene mutations identified by the MutSig algorithm which recognized point mutations or small indels, copy number variants or epigenetic effects like methylation were not considered in this study. Consequently, the identified results mainly reflect the influences caused by the point mutations or small indels of the genes. In the future, we will design new workflows to further explore the impacts caused by the other types of alterations that were not included here.

## Conclusions

Here, we obtained a comprehensive description on the key prognosis-relevant genes of *KEAP1*/*NFE2L2*/*CUL3* for LUSC. The interesting molecular and clinical patterns between the two subtypes in LUSC reinforce the highly heterogeneity of LUSC. Although large-scale genomic studies have identified the frequent mutations in KEAP1/NRF2/ CUL3 pathway and their prominent roles in LUSC and other cancers, less attention is paid on their complicated down-stream effects. Also, an integrative study of both SMG and the down-stream expression profiles of LUSC help recognized two robust subtypes where one subtype with markedly suppressed expressions in EMT and inflammatory response pathways showed significant better survivals and higher mutation frequencies in the KEAP1/NRF2/ CUL3 pathway. This alternative LUSC subtyping can provide promising diagnosis references and potential therapeutic targets for LUSC.

## Supplementary information

**Additional file 1: Figure S1.** Expressional alterations and clinical significance of the SMGs. A. Boxplots of the expressions of SMGs in mutated and wild type tissues. B. Km-plots of SMGs with significant impacts on LUSC. **Figure S2.** Top-ranked differentially expressed genes between samples with and without certain mutations. SMGs (yellow nodes) and their relevant differentially expressed genes are linked by edges. The colors of the down-stream genes represent their roles in cancer as annotated in COSMIC database. Red and green edge colors respectively represent positive and negative correlations, and the edge width is proportional to the absolute value of log2FC. **Figure S3.** Survival analysis about the subtype relevant SMGs. A-D. KM-plots about the survival curves of patients with and without mutations in *NFE2L2* (A), *KEAP1* (B), *CUL3* (C) and *COL11A1* (D). The differences in survival rates were tested by log rank test.

## Data Availability

The data that support the findings of this study are available from GDC ((https://gdc.cancer.gov/about-data/publications/pancanatlas)) and GEO (https://www.ncbi.nlm.nih.gov/geo/query/acc.cgi?acc=GSE30219;https://www.ncbi.nlm.nih.gov/geo/query/acc.cgi?acc=GSE37745).

## Abbreviations

COSMIC: Catalogue of somatic mutations in cancer
Coxph: Cox proportional hazards regression
EMT: Epithelial mesenchymal transition
GDC: Genomic data commons
GEO: Gene expression omnibus
GSEA: Gene set enrichment analysis
LUSC: Lung squamous cell carcinoma
LUAD: Lung adenocarcinoma
maf: Mutation annotation format
SMG: Somatic mutated gene
TCGA: The cancer genome atlas
