# Supplementary figures and images for: An integrative investigation on significant mutations and their down-stream pathways in lung squamous cell carcinoma reveals CUL3/KEAP1/NRF2 relevant subtypes
Source: Mol Med. 2020 May 20;26:48. doi: 10.1186/s10020-020-00166-2 (PMC7240936; doi:10.1186/s10020-020-00166-2)

A

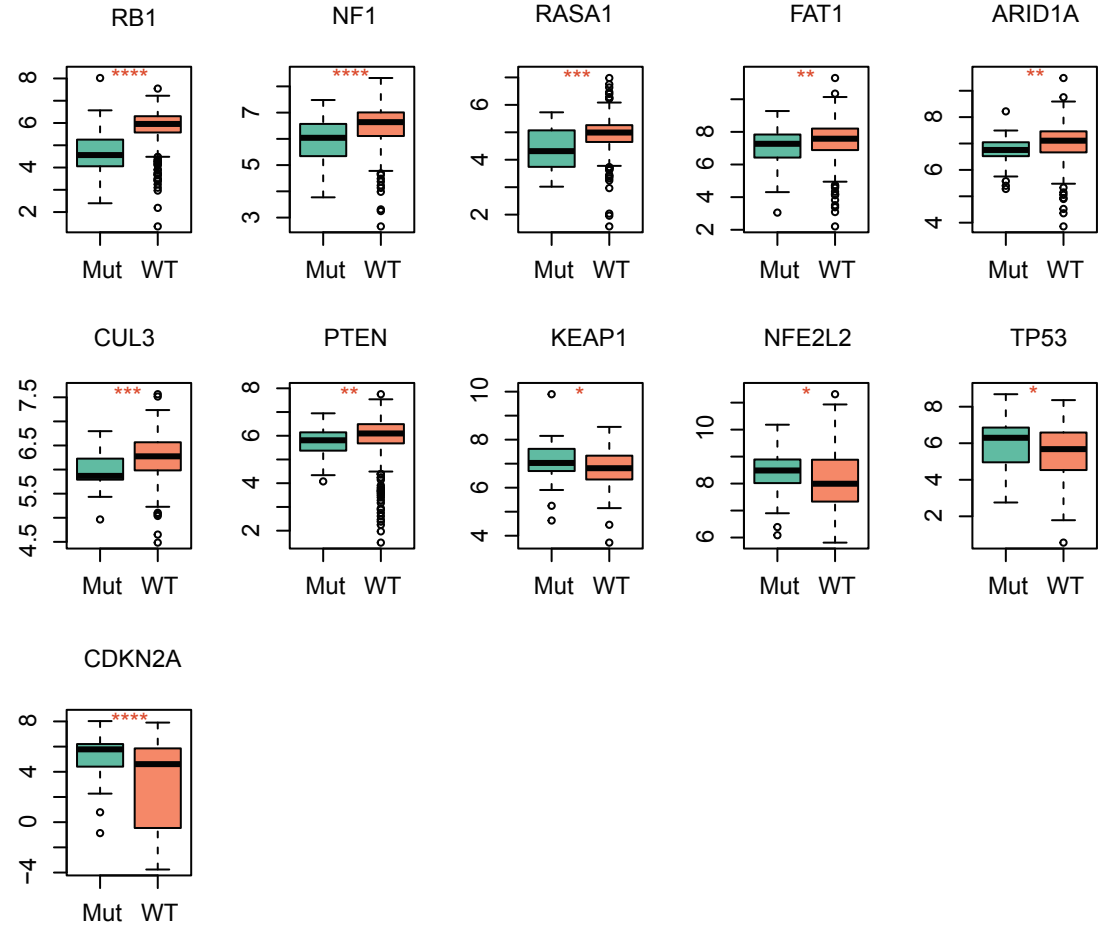

B

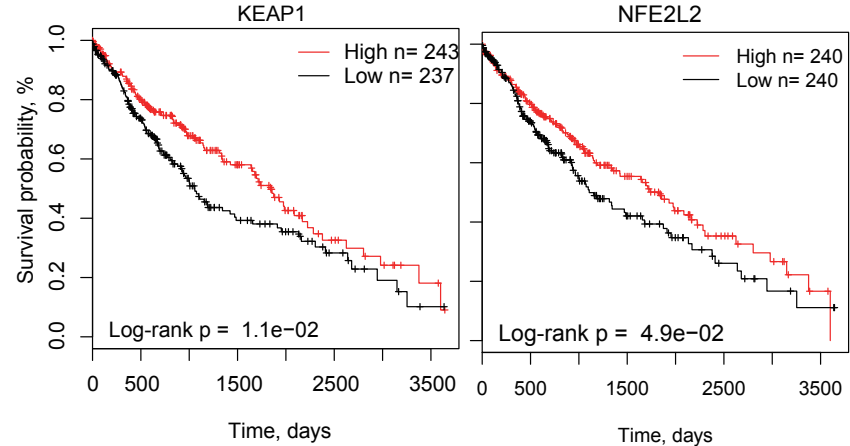

Figure S1

## Role for downstream genes

- Oncogene
- Tumor suppressor gene
- Unclear

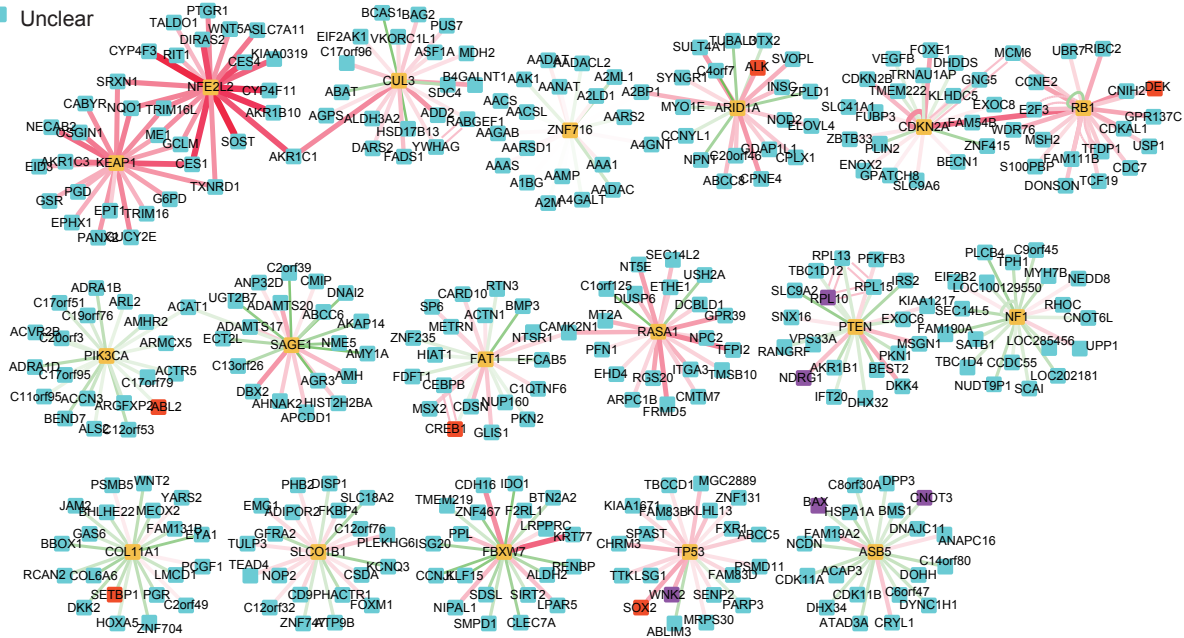

### Figure S2

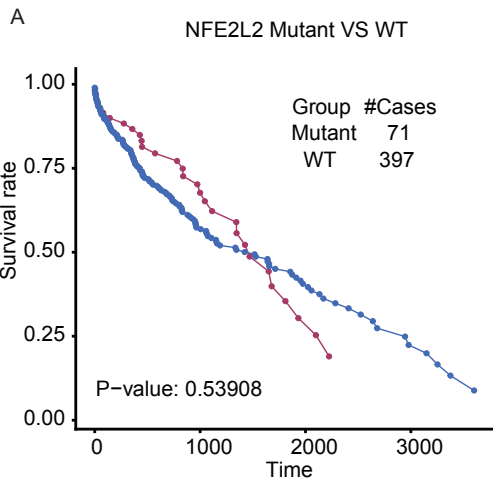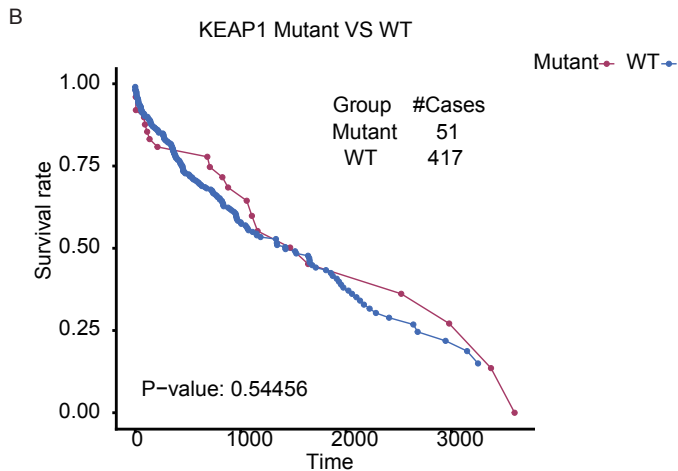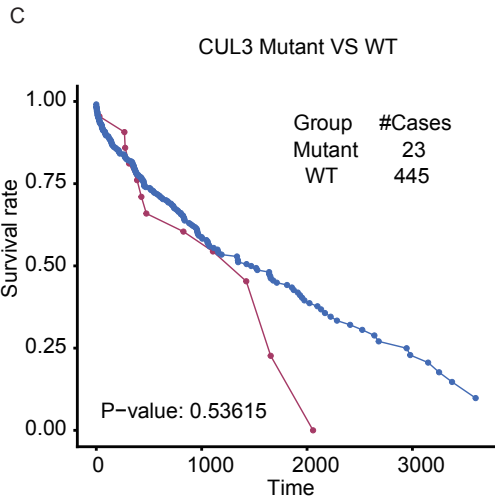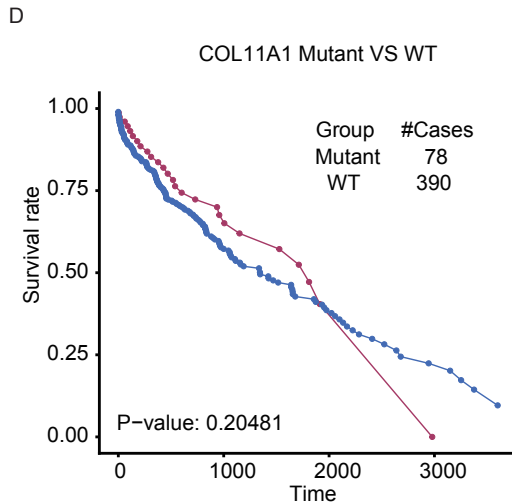

**Figure S3**

Supplement: Supplementary file 1 — Additional file 1: Figure S1. Expressional alterations and clinical significance of the SMGs. A. Boxplots of the expressions of SMGs in mutated and wild type tissues. B. Km-plots of SMGs with significant impacts on LUSC. Figure S2. Top-ranked differentially expressed genes between samples with and without certain mutations. SMGs (yellow nodes) and their relevant differentially expressed genes are linked by edges. The colors of the down-stream genes represent their roles in cancer as annotated in COSMIC database. Red and green edge colors respectively represent positive and negative correlations, and the edge width is proportional to the absolute value of log2FC. Figure S3. Survival analysis about the subtype relevant SMGs. A-D. KM-plots about the survival curves of patients with and without mutations in NFE2L2 (A), KEAP1 (B), CUL3 (C) and COL11A1 (D). The differences in survival rates were tested by log rank test. [file 10020_2020_166_MOESM1_ESM.pdf]
